# Deletion of Integron-Associated Gene Cassettes Impact on the Surface Properties of *Vibrio rotiferianus* DAT722

**DOI:** 10.1371/journal.pone.0058430

**Published:** 2013-03-06

**Authors:** Rita A. Rapa, Ronald Shimmon, Steven P. Djordjevic, H. W. Stokes, Maurizio Labbate

**Affiliations:** 1 The ithree Institute, University of Technology, Sydney, Australia; 2 Chemical Technology and Forensic Science, University of Technology, Sydney, Australia; Universite Libre de Bruxelles, Belgium

## Abstract

**Background:**

The integron is a genetic recombination system that catalyses the acquisition of genes on mobilisable elements called gene cassettes. In *Vibrio* species, multiple acquired gene cassettes form a cassette array that can comprise 1–3% of the bacterial genome. Since 75% of these gene cassettes contain genes encoding proteins of uncharacterised function, how the integron has driven adaptation and evolution in *Vibrio* species remains largely unknown. A feature of cassette arrays is the presence of large indels. Using *Vibrio rotiferianus* DAT722 as a model organism, the aim of this study was to determine how large cassette deletions affect vibrio physiology with a view to improving understanding into how cassette arrays influence bacterial host adaptation and evolution.

**Methodology/Principal Findings:**

Biological assays and proteomic techniques were utilised to determine how artificially engineered deletions in the cassette array of *V. rotiferianus* DAT722 affected cell physiology. Multiple phenotypes were identified including changes to growth and expression of outer membrane porins/proteins and metabolic proteins. Furthermore, the deletions altered cell surface polysaccharide with Proton Nuclear Magnetic Resonance on whole cell polysaccharide identifying changes in the carbohydrate ring proton region indicating that gene cassette products may decorate host cell polysaccharide via the addition or removal of functional groups.

**Conclusions/Significance:**

From this study, it was concluded that deletion of gene cassettes had a subtle effect on bacterial metabolism but altered host surface polysaccharide. Deletion (and most likely rearrangement and acquisition) of gene cassettes may provide the bacterium with a mechanism to alter its surface properties, thus impacting on phenotypes such as biofilm formation. Biofilm formation was shown to be altered in one of the deletion mutants used in this study. Reworking surface properties may provide an advantage to the bacterium’s interactions with organisms such as bacteriophage, protozoan grazers or crustaceans.

## Introduction

Integrons are genetic elements that include site-specific recombination functions. They integrate and express genes present on mobilisable elements called gene cassettes. The integron consists of three components, a gene (*intI*) encoding an integrase, an attachment site (*attI*) where gene cassettes insert and a promoter (P_c_) adjacent to *attI* that drives transcription of inserted gene cassettes [1], [2]. Numerous classes of integrons have been identified and these classes are defined by the sequence that encodes the integrase [3]. First discovered in clinical settings, class 1 integrons are commonly found on resistance plasmids within pathogens and commensals. They carry small cassette arrays of, commonly, 1–6 gene cassettes and are a major contributor to the problem of antibiotic resistance [4]. In the natural environment, the integron is present in chromosomal locations with approximately 10% of sequenced genomes containing chromosomal integrons [5]. In these organisms, cassette arrays can vary substantially in size (0 – >200 cassettes) and rarely carry known antibiotic resistance gene cassettes. Given this, integrons are regarded as having a more general role in evolution than simply carrying and expressing antibiotic resistance genes [1], [5].

*Vibrio* species are free-living marine bacteria that carry out diverse roles and occupy a wide range of niches in association with higher organisms. They can be found in symbiotic or pathogenic relationships with a wide variety of marine hosts such as prawns, coral, fish, invertebrates, plants and marine mammals [6]. One of the major drivers of the diversification of *Vibrio* species is lateral gene transfer (LGT) [7]. *Vibrio* species carry particularly large cassette arrays with the integron and associated cassettes making up 1–3% of the entire bacterial genome and as such is a substantial source of laterally acquired DNA in vibrios. How the integron influences the evolution of *Vibrio* species remains largely unknown although recent studies have provided new insight into the biology of integrons. The SOS response induces the integron-integrase resulting in enhanced rates of acquisition, deletion and movement of gene cassettes across the array [8]. This suggests that the bacterial host uses the integron as a mechanism for adaptation to stressful environments. Furthermore, in *V. rotiferianus* DAT722, the majority of the 116-gene cassettes in the array are transcribed, with different cassettes transcribed in response to differing growth conditions [9]. These data indicate the presence of diverse promoters other than P_c_ within the array and demonstrates that almost all gene cassettes are able to add to the adaptive potential of the cell.

Approximately 75% of gene cassettes in *Vibrio* species encode proteins that are of unknown function [5] although a handful have been characterised and show to produce functional proteins [2], [10], [11], [12], [13], [14]. In a recent study, mutants with deletions in the integron cassette array of *V. rotiferianus* DAT722 were created to assist in identifying phenotypes for uncharacterised gene cassettes. In this study, it was shown that deletion of a specific group of cassettes substantially impacted growth and porin regulation demonstrating that apart from providing accessory functions, gene cassette products can integrate into complex regulatory pathways [15]. A feature of cassette arrays is that large groups of contiguous cassettes can be deleted from an array at a given time [16]. For example, a large 38 gene cassette deletion was found in some strains of pathogenic *V. cholerae* O1 El Tor strains but not in others with the deletion being the only known difference between these strains [16]. How this impacts the bacterial cell and its survivability in the environment is unknown.

In an effort to understand how deletion of contiguous gene cassettes might affect vibrio adaptation and evolution, we used physiological growth, stress assays, proteomic and chemistry-based techniques to characterise how engineered deletions of gene cassettes in *V. rotiferianus* DAT722 affects vibrio physiology. We show that deletion of gene cassettes affects surface structures of the bacterial cell, specifically, properties of bacterial polysaccharide. We hypothesise that acquisition, movement or deletion of some gene cassettes within the array might provide the host organism with a mechanism for altering surface properties. In this study, we show that deletion of gene cassettes can alter biofilm formation and hypothesise that modifying surface properties may also have implications for how vibrios interact with bacteriophage, protozoan grazers and higher marine organisms.

## Results

In this study, we compared the physiological effects of deleting multiple genes cassettes from the cassette array of *V. rotiferianus* DAT722. Construction and description of the mutants is described in the Materials and Methods (strains used in this study are shown in Table 1). Deletion mutants d16-60, d50-60 and d72-92 have had gene cassettes 16–60, 50–60 and 72–92 deleted from the 116 gene cassette array respectively. A table providing details on the deleted cassettes is included as supplementary material (Table S1).

**Table 1:** List of strains and plasmids.

Strain or plasmid	Relevant genotype^1^	Reference
*Vibrio rotiferianus* DAT722	wild-type	[43]
DAT722-Sm	DAT722; spontaneous Sm^R^ mutant	[15]
MD7	DAT722-Sm; Single recombination cross-over of pMAQ1081 into cassette 61, Km^R^	[15]
SC-8B61	DAT722-Sm; Single recombination cross-over of pMAQ1081 into cassette 61, Km^R^	This study
SC-8A91	DAT722-Sm; Single recombination cross-over of pMAQ1081 into cassette 93, Km^R^	This study
d16-60	DAT722-Sm; Δcassettes 16-60, Sm^R^, Km^R^	[15]
d16–60a	DAT722-Sm; Δcassettes 16–60, Sm^R^, Km^R^	This study
d50–60	DAT722-Sm; Δcassettes 50-60, Sm^R^, Km^R^	This study
d72–92	DAT722-Sm; Δcassettes 72–92, Sm^R^, Km^R^	This study
d72–92a	DAT722-Sm; Δcassettes 72–92, Sm^R^, Km^R^	This study

1 Sm^R^, streptomycin resistance; Km^R^, kanamycin resistance.

### Does Deletion of Gene Cassettes Affect Growth?

**Figure 1:**
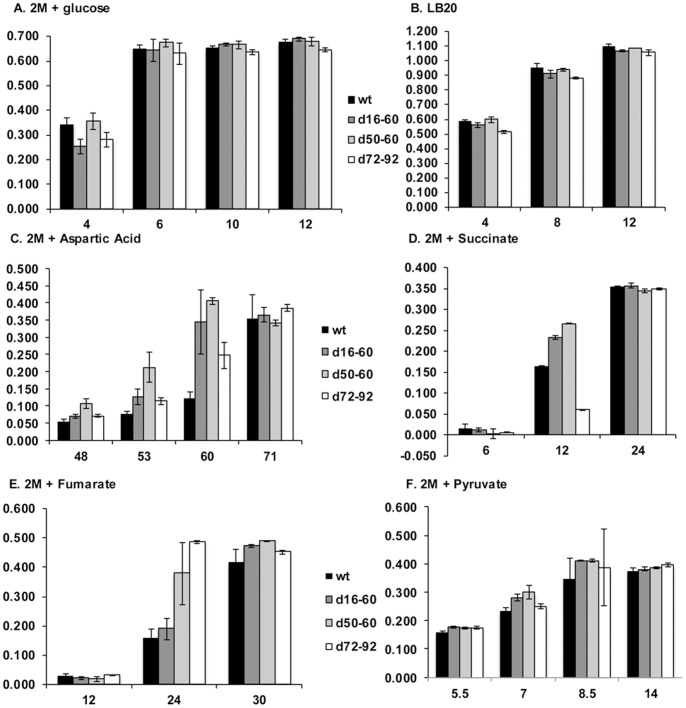
Growth curves. Growth of wt and deletion mutants in LB20 and 2M salts+differing carbon sources. Indicated on the horizontal axis are time intervals (hrs) where differences in growth between wt and deletion mutants were identified. Error bars show statistical significance of triplicate optical density readings.

Comparison growth curves (Figure 1) of the parent and isogenic deletion mutants were conducted to determine whether the deletion of gene cassettes affected growth. Growth curves were conducted in LB20 and in 2M salts+various carbon sources including glucose, fumarate, succinate, aspartic acid and pyruvate. On a logarithmic scale, growth of the wild-type (wt) and deletion mutants did not reveal any obvious changes in lag phase or growth rate (data not shown). However, minor reproducible changes were observed in optical density when growth of the deletion mutants on 2M+various carbon sources was compared to the wild-type (wt) parent. Specifically, the wt had at least 2-fold less cells when grown in 2M+aspartic acid compared to d16–60, d50–60 and d72–92 at 60 hours growth (Figure 1C). In 2M+succinate, d72–92 had approximately 3-fold less cells at 12 hours growth when compared to the wt and other deletion mutants (Figure 1D). In 2M+fumarate d50–60 and d72–92 grew faster than the wt and d16–60 mutant with the d72–92 having approximately 3-fold more cells than the wt at 24 hours (Figure 1E). In 2M+pyruvate d16–60 and d50–60 have higher optical density readings at after 7 and 8.5 hours (Figure 1F).

These data indicate one or more of multiple possibilities. The deletions have altered the metabolic balance within the cell resulting in minor variation in growth, the deletions have removed cassettes involved in stress upon nutrient deprivation or that permeability of the cells has been changed altering the uptake of the carbon source or other nutrients. While the deletions did not appear to have affected final cell densities, the minor variations in growth could affect competitiveness in the environment.

### Gene Cassettes do not Affect Environmental Stress Survival

**Figure 2:**
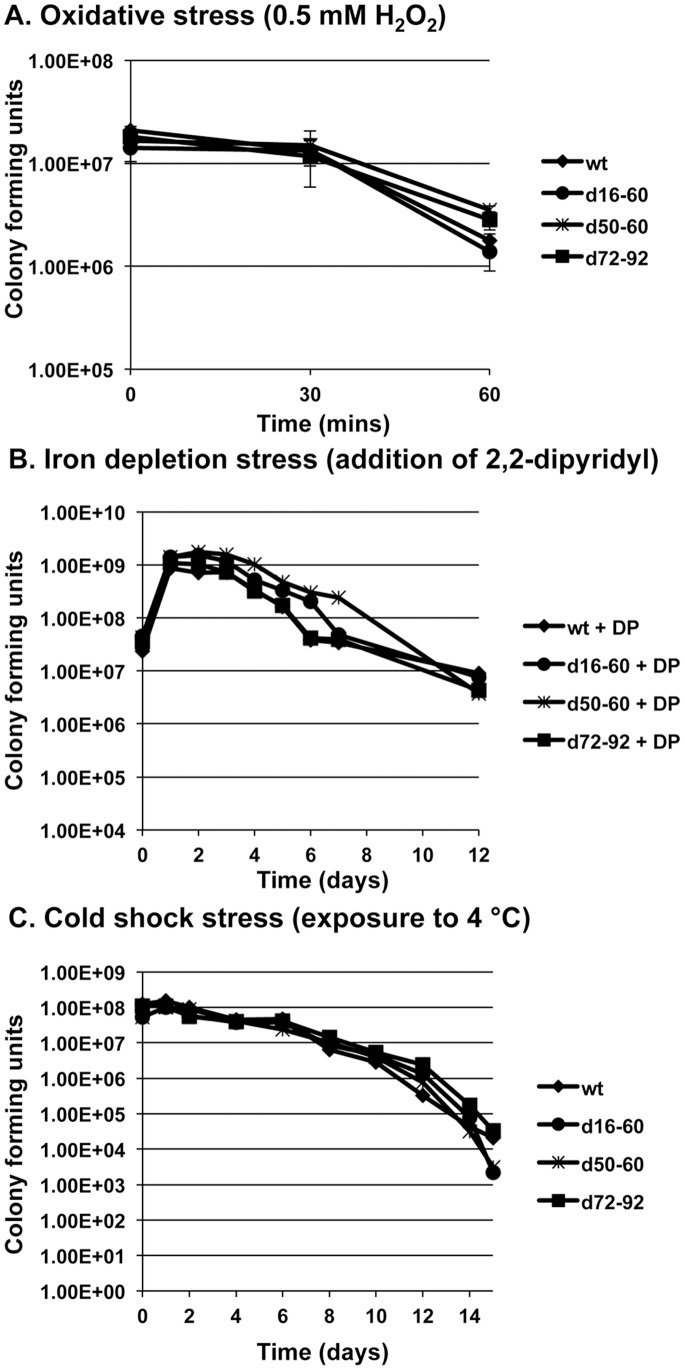
Environmental stress assays. Environmental stress assays showing oxidative (A), iron depletion (B) and cold shock stress (C). The figures shown here are representative of three independent experiments.

Recent studies have shown that SOS-inducing stress activates the integron-integrase gene and subsequent gene cassette shuffling [8]. As a result, it has been suggested that in times of stress, gene cassette shuffling may be a mechanism for surviving environmental stress. To determine whether this is the case, the wt parent and deletion mutants were subject to stress conditions that the organism might encounter in the natural environment including oxidative, iron depletion and cold shock stresses (Figure 2). For each tested stress, no major difference was observed between the deletion mutants and wt indicating that at least for the deleted gene cassettes, there is no specific role in environmental stress.

### How does Deletion of Gene Cassettes Affect Whole Cell Protein Regulation and Secretion of Proteins?

Gene cassettes can have adverse effects on growth indicating that mobile genes integrate into host cellular networks [15]. Thus, we were interested in how a large gene cassette deletion, not adversely affecting growth rates would affect host whole cell physiology. It was hypothesised that deletion of cassettes, thereby resulting in the loss of some proteins, would result in changes to cell networks. Identifying these changes may provide further insight into gene cassette function and the influence mobile genes have on the adaptive potential of the cell.

Mutant d16–60 (the largest array deletion) was subjected to 2D-PAGE and compared to the wt when grown under complete (LB20) and minimal growth conditions (2M+glucose). Furthermore, protein expression at stationary and mid-logarithmic phases was compared under both nutrient conditions. For cells grown in LB20 to stationary phase, two out of 325 protein spots were shown to be differentially expressed between wt and d16–60, in mid-logarithmic phase, four out of 357 were differentially expressed (Table 2). For cells grown in minimal media to stationary phase, three out of 360 protein spots were differentially expressed and in mid-logarithmic phase, three out of 201 were differentially expressed (Table 3). Thus, approximately 0.5–1% of protein spots identified between wt and d16–60 were at least 2-fold differentially expressed with a maximum fold difference of 3.7 for spot 2CM (Table 2). This analysis showed quite subtle differences in the whole cell proteome between wt and d16–60 in the conditions tested, indicating only a minor effect of the 46 cassette deletion (∼31 kb) in the expression of detectable high abundance proteins in the proteome. It is possible that changes to lower abundance proteins not detected using this methodology is occurring.

**Table 2:** Differentially expressed spots between deletion mutant d16–60 mutant and wild-type *Vibrio rotiferianus* DAT722 in LB20.

Growth Phase	Differentially expressed spot	LC-MS/MS match(s)	Number of matched peptides		PEAKS score* (%)	Fold changein mutant	Accessionnumber
***Mid-logarithmic phase***							
	1CM	OmpA; VrotD_16305		8	99.0	−2.31	ZP_08911638
	2CM	OmpU-like outer membrane protein		10	98.8	−3.68	ZP_08912594
	∧3CM	50S ribosomal protein L9		10	98.8	−2.61	ZP_08908422
		OmpA-like membrane protein; VrotD_08232		7	97.5		ZP_08910036
	∧4CM	ATP-dependent Clp protease proteolytic subunit		9	98.3	−2.90	ZP_08909655
		S-ribosylhomocysteinase/Autoinducer-2 production protein LuxS		5	97.9		ZP_08911325
		Type VI secretion-related protein		3	96.8		ZP_08909611
		Shikimate kinase I		3	96.4		ZP_08908430
***Stationary phase***							
	1CS	Unknown protein (gene is surrounded by genes encoding O-antigen biosynthesis or export); VrotD_02720		15	99.0	+2.30	ZP_08908944
	2CS	Nitrogen regulatory protein P-II		13	96.9	−2.75	ZP_08911087

* highest PEAKS score (percentage based on a p-value <0.05) was taken as the closest peptide match

∧ denotes co-migrating protein spots.

**Table 3:** Differentially expressed spots between deletion mutant d16–60 mutant and wild-type *Vibrio rotiferianus* DAT722 in 2M+glucose.

Growth Phase	Differentially expressed spot	LC-MS/MS match(s)	Number of matched peptides	PEAKS score* (%)	Fold changein mutant	Accessionnumber
***Mid-logarithmic phase***						
	∧1MM	Putative membrane protein; VrotD_04538	6	98.6	+2.73	ZP_08909304
		Phosphoribosylformimino-5-aminoimidazole carboxamide ribotide isomerase (Histidine biosynthesis)	4	95.1		ZP_08910780
	2MM	Alkyl hydroperoxide reductase subunit C-like protein	3	98.3	+2.45	ZP_08909276
	3MM	6,7-dimethyl-8-ribityllumazine synthase	15	99.0	−2.01	ZP_08909366
***Stationary phase***						
	∧1MS	Cysteine synthase A	12	95.4	+2.11	ZP_08909513
		Acetyl-coenzyme A carboxyl transferase alpha chain	7	92.0		ZP_08911090
		OmpT	17	90.2		ZP_08909742
	2MS	Unknown protein; VrotD_21668	10	98.3	−2.00	ZP_08912704
	∧3MS	Unknown protein; VrotD_07347	16	96.1	+2.55	ZP_08909861
		NAD-dependent glyceraldehyde-3-phosphate dehydrogenase	13	96.0		ZP_08910944
		Phenylalanyl-tRNA synthetase alpha chain	9	94.4		ZP_08910491
		Universal stress protein E	10	93.8		ZP_08910109

* highest PEAKS score (percentage based on a p-value <0.05) was taken as the closest peptide match.

∧ denotes co-migrating protein spots.

Due to co-migration of proteins not all protein spots could be unambiguously identified. Out of the unambiguous protein spots that were differentially expressed, surface-associated proteins were identified (Tables 2 and 3), including OmpA (spot 1CM), an OmpU-like protein (spot 2CM) and an unknown protein (spot 1CS). Since the gene that encodes this unknown protein is located in a region of the genome responsible for polysaccharide biosynthesis and PSORTb analysis shows its localisation to be extracellular [17], the unknown protein is likely be involved in polysaccharide synthesis (Table S2). Metabolic proteins were also differentially expressed including proteins with homology to nitrogen regulatory protein P-II (spot 2CS), alkyl hydroperoxide reductase subunit C-like protein (spot 2MM) and 6,7-dimethyl-8-ribityllumazine synthase (spot 3MM). An uncharacterised protein (spot 2MS) was also identified as differentially expressed. No gene cassette proteins were identified as missing in the d16-60 mutant even though most gene cassettes in *V. rotiferianus* DAT722 are transcribed [9]. Presumably this is due to high abundance proteins masking the presence of lower abundance proteins [18], [19].

**Figure 3:**
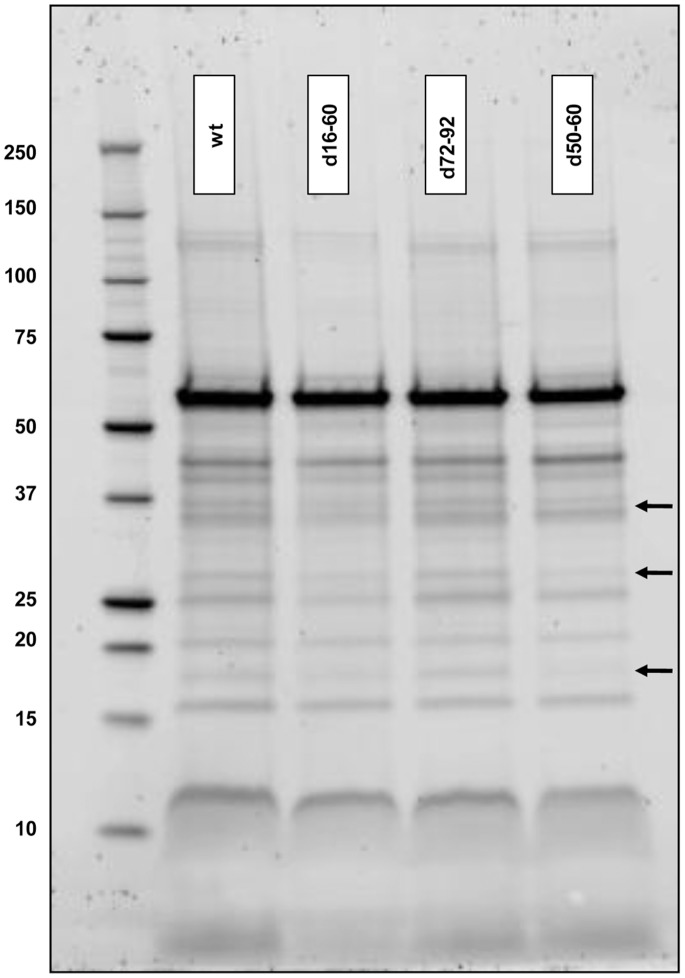
Gel electrophoresis of supernatant proteins. Gel electrophoresis of precipitated supernatant proteins from *V. rotiferianus* DAT722 (wt) and deletion mutants grown in 2M+glucose. Protein standards indicated left of the gel have sizes in kDa. Lanes are labeled with strain names and arrows indicate protein bands of differing abundance.

In addition to 2D-PAGE, we compared the secreted proteins of wt with all deletion mutants from cells grown in 2M+glucose and found no major differences in terms of presence or absence of protein bands. However, slight variations in the abundance of some proteins were observed and are labelled in Figure 3.

### Congo Red Staining of Bacterial Colonies Show Differences in All Deletion Mutants

During 2D-PAGE analysis, it was observed that protein extracted from stationary phase d16–60 cells grown in 2M+glucose consistently contained a substance that interfered with the isoelectric focusing (IEF) step in 2D-PAGE (Figure S1). This substance was removed from the sample by washing cells with 2% NaCl pre-protein extraction and represents a point of physiological difference between wt and the d16–60 mutant. Although this substance is yet to be identified, it is extracellular and weakly associated with the cell surface.

Multiple contaminating substances can interfere with the IEF step including DNA, cell wall material and polysaccharides [19]. Since the substance specific to d16–60 is extracellular in nature and a protein putatively involved in polysaccharide biosynthesis was identified by the 2D-PAGE analysis (spot 2CS), we hypothesised the contaminating substance was polysaccharide in nature.

**Figure 4:**
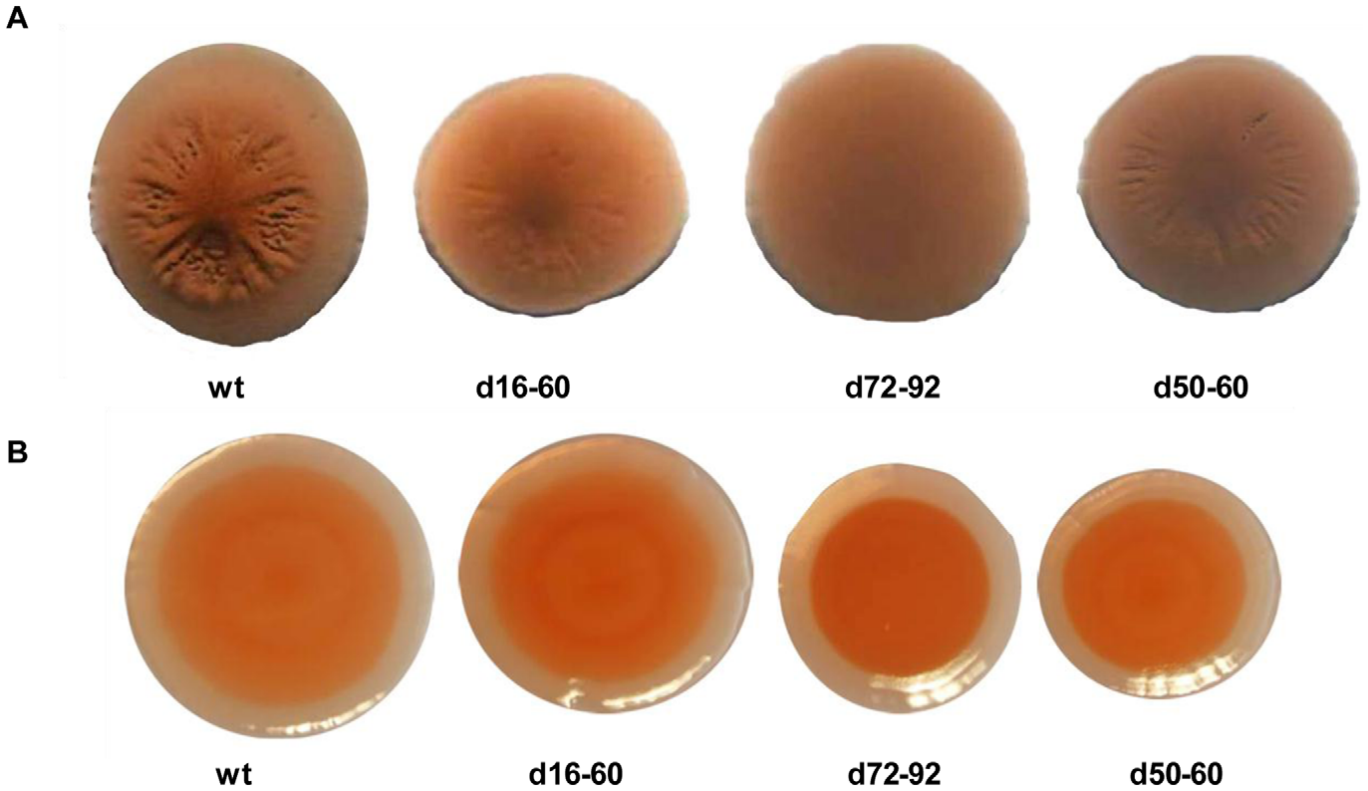
Congo red colony morphology. Colonies of *V. rotiferianus* DAT722 and deletion mutants grown on agar plates supplemented with 0.001% congo red. Colonies grown on LB20 and 2M +0.2% glucose supplemented with congo red are shown in panels A and B respectively. All colonies were imaged after 7 days growth at 28°C.

To confirm this, we used congo red staining to determine whether deleting cassettes affected colony wrinkling in all deletion mutants. Colony wrinkling has previously been shown to be associated with the extracellular/capsular polysaccharide in vibrios [20]. Interestingly, no differences could be observed between the wt and deletion mutants on 2M+glucose medium especially since it was in this medium that d16–60 was producing the contaminating substance (Figure 4). However, substantial changes in colony wrinkling were observed when cells were grown on LB20. On LB20, wt colonies showed repeated characteristic wrinkling architecture whereas the deletion mutants showed differing levels of wrinkling indicating a change in the amount or structure of produced polysaccharide in these mutants. The lack of wrinkling of colonies when grown on 2M+glucose medium indicates that *V. rotiferianus* DAT722 produces different polysaccharide(s) when exposed to different growth conditions that does not bind congo red. This production of different polysaccharides under different growth conditions has been reported in other bacteria [21], [22].

### Proton NMR Spectroscopy of Whole Cell Polysaccharides

To confirm that deletion of gene cassettes had modified cell surface polysaccharides, total polysaccharide from wt, d16–60 and d72–92 was extracted from cells grown in 2M+glucose medium using a hot phenol extraction method (Material and Methods) and subjected to preliminary ^1^H NMR analysis. These two mutants were selected for ^1^H NMR due to the different cassettes deleted. To ensure any changes were a result of the deletions and not any secondary mutation(s), total whole cell polysaccharide was also extracted and purified from identical but independently derived mutants: d16–60a and d72–92a.

Whole cell polysaccharide extractions include, lipopolysaccharide (LPS; made up of the O-antigen, core polysaccharide and lipid A core) and capsular/extracellular (C/EPS) polysaccharide structures, so any changes are related directly to these structures. However which polysaccharide moiety being altered cannot be identified.

**Figure 5:**
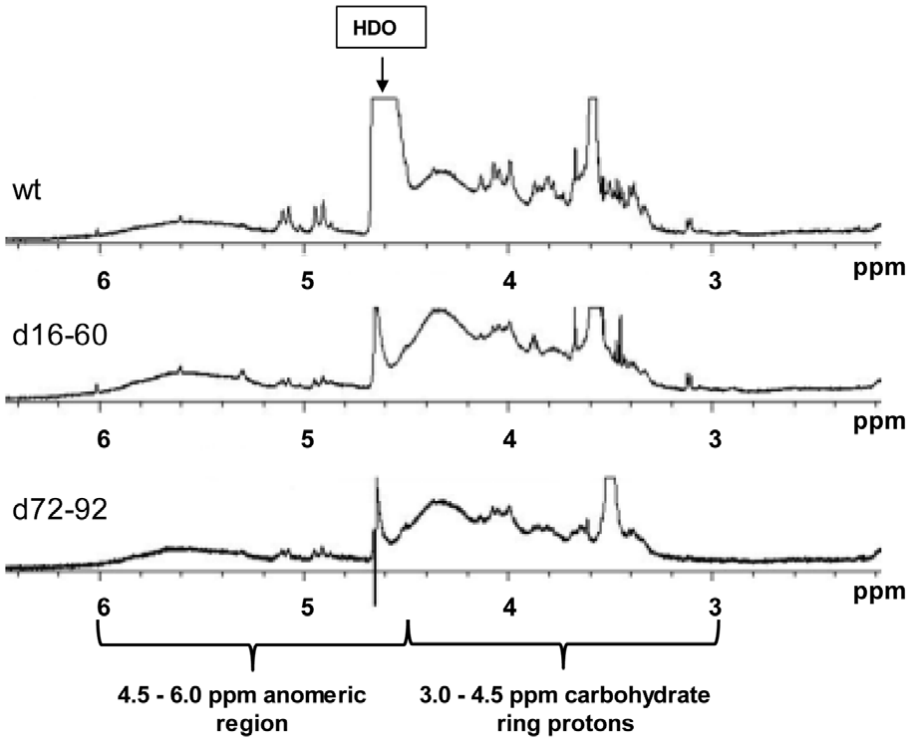
Water suppression H^1^ NMR spectra. H^1^ NMR spectra of wt, d16–60 and d72–92 whole cell polysaccharide.

We initially compared the ^1^H NMR scans of the wt with d16–60 and d72–92 (Figure 5). The scans showed dissimilarity between the wt and both d16–60 and d72–92, as well as dissimilarity between the two deletion mutants themselves in the chemical shift region of approximately 3.0–4.5 ppm (Figure 5). Fresh extractions of the wt, d16–60a and d72–92a produced ^1^H NMR scans showed similar scans to the first batch however, due to a small modification in the extraction methodology (see Materials and Methods) differences in purity were observed. Nevertheless, changes were again observed in the chemical shift region of approximately 3.0–4.5 ppm (Figure S2). Therefore, these changes to the whole cell polysaccharide are confirmed to be a direct result of the gene cassette deletions.

The 3.0–4.5 ppm region is identified as the carbohydrate ring proton region of the diagnostic NMR spectrum. Alterations in the chemical shift between wt and deletion mutants indicate that there are changes in the functional groups attached to carbohydrate molecules. A peak labelled HDO (deuterated water) is present at 4.75 ppm (Figure 5 and Figure S2). Differences in the width of this peak indicated a saturated water signal as a consequence of incomplete exchange of all hydrogen atoms present in water with deuterium (see Materials and Methods). This does not affect results presented. The anomeric region labelled in Figure 5 shows little change in the chemical shift of peaks between wt and mutants indicating that there are no alterations in the type of sugar (i.e. glucose, sucrose, fructose) present along the backbone of the polysaccharide structure(s).

Overall, it is not surprising that there are distinct differences between the d16–60 and d72–92 mutants as these contain different gene cassette deletions across the array. To elucidate exactly how the deletions have altered the polysaccharide(s) from *V. rotiferianus* DAT722, further comprehensive chemical characterisation of the polysaccharides is required. Our preliminary analyses has shown that there are at least two polysaccharide structures are produced by *V. rotiferianus* DAT722 (data not shown) and these would need to be separated, purified and the structure determined for each polysaccharide.

### A Gene Cassette Deletion Affects Biofilm Formation

**Figure 6:**
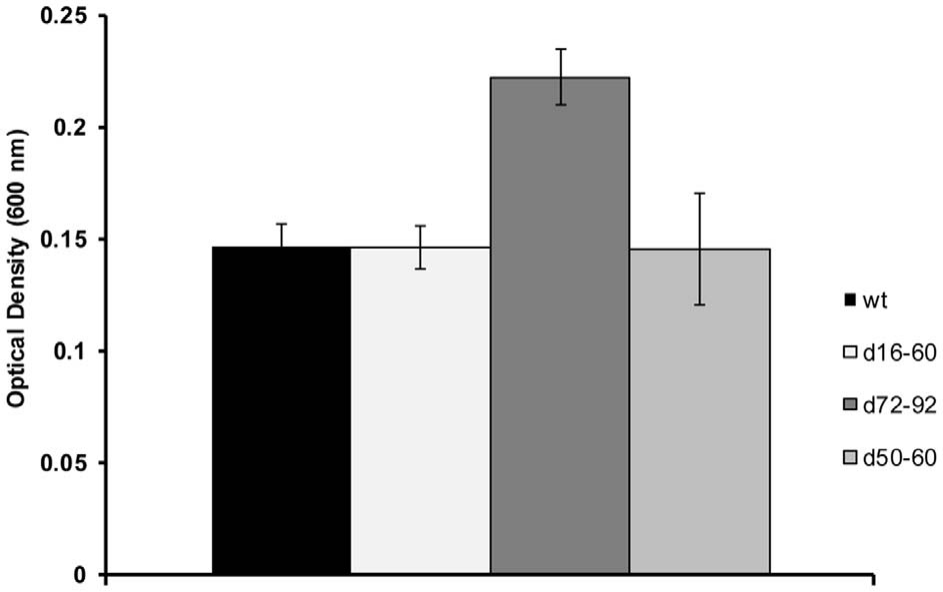
Graph showing optical density of crystal violet stained cells. Bar graphs showing significantly higher adhesion of d72–92 indicated by the asterisk.

As alterations to bacterial surface polysaccharide can affect how the bacterial cell interacts with the environment, studies were performed to identify whether the deletion of cassettes impacted on biofilm formation. Deletion mutant d72–92 showed statistically significant higher biomass when compared to the wt, d16–60 and d50–60 when stained with crystal violet (Figure 6). It is likely that the changes in polysaccharide as a result of the 72–92 deletion, has altered the organism’s ability to adhere or form a biofilm. Biofilm assays were carried out on a hydrophilic plastic surface however, different affects might be observed in the deletion mutants if adhesion or biofilm experiments were conducted on other surfaces such as metal, chitin or eukaryotic tissues.

## Discussion

The integron/gene cassette system was first identified as a consequence of its contribution to the acquisition by bacteria of antibiotic resistance genes [23]. In clinical isolates, class 1 integrons have accumulated diverse tandem arrays of gene cassettes, most of which encode antibiotic resistance functions [24] driven by the strong selection imposed by the broad use of antibiotics by humans over the last 70 years. However, integrons and gene cassettes are ancient structures and the mobile gene cassette metagenome represents a vast reservoir of novel genes [1], [5], [25], [26], [27]. While the majority of the predicted gene products have no known function, environmental surveys strongly imply they are adaptive and where examined, structural and other studies support this notion [28], [29], [30].

Using *Vibrio rotiferianus* strain DAT722 as a model, we have recently shown that deletion of cassettes 8–60 produced mutants with a substantial loss of growth fitness [15]. Furthermore, this large deletion could not be supported without a suppressor mutation. This study demonstrated that recently acquired gene cassettes (or any mobile DNA) have the capacity to be rapidly integrated into pivotal cell networks [15]. This is in contrast to the generally held view that gene cassettes only impart accessory roles (e.g. antibiotic degradation), an issue we recently explored [31]. Following on from this study, it was of interest to determine how gene cassettes affect bacterial physiology in the absence of any detrimental growth defect. In contrast to d8–60, deletion of cassettes 16–60 (d16–60) had a healthy growth phenotype [15] and thus we utilised 2D-PAGE to determine how the large 46 cassette deletion altered protein physiology. Given the size of the deletion (∼1/3 of the array) only 0.5–1% of the total proteome was differentially expressed in d16–60 with fold changes at approximately the 2-fold range (maximum change 3.6 fold). These changes were consistent across two different media (complete and minimal) and two different growth phases (mid-logarithmic and stationary). Since 2D-PAGE detects only the most abundant proteins in the cell, it can be stated that the deletion has not affected the major metabolic pathways of the cell. However, it is possible that there is a higher degree of change to lower abundance proteins not detected here. Nevertheless, in conjunction with the secreteome analysis, these data indicate that deletion of cassettes 16–60 has not adversely impacted on the major cellular pathways of the cell in contrast to our previous study with mutant d8–60 [15]. Furthermore, d16–60 and all other deletion mutants described in this study were not disadvantaged in environmental stress assays including oxidative stress, iron stress and cold stress. These data combined indicate that the majority of gene cassettes are maintained independently of host cell networks and are most likely not involved in stress adaptation.

During our analyses, evidence emerged that the gene cassette deletions alter host polysaccharide. This was confirmed with congo red staining and ^1^H NMR analysis of whole cell purified polysaccharide. Multiple polysaccharide structures can exist on the Gram negative cell surface including lipopolysaccharide (LPS), a polysaccharide covalently linked to a lipid (lipid A core) that is embedded in the membrane and capsular/extracellular polysaccharide (C/EPS), a polysaccharide closely associated with the cell surface. From our preliminary ^1^H NMR analyses, changes between the wt and both d16–60 and d72–92 mutants, as well as dissimilarities between the two mutants themselves was identified in the carbohydrate ring proton region. This indicates that gene cassette-associated products are most likely influencing functional groups linked to the sugar component of polysaccharide structure(s). For example, gene cassette products could be adding/removing functional groups such as NH_2_ or CH_3_ groups. The biological ramifications for such changes are substantial since they can affect processes such as bacterial-host interactions or virulence. For example, a recent study determined that the polysaccharide component of LPS plays a critical role in the colonisation of the light organ of squid species *Euprymna scolopes* [32]. CPS is also widely known to be important in virulence including a pandemic strain of *V. parahaemolyticus* [20]. Changes to surface polysaccharide are also likely to affect resistance to bacteriophage and biofilm formation [33]. We showed that as a consequence of deleting 22 cassettes to produce mutant d72–92, biofilm formation to a plastic hydrophilic surface was significantly increased when compared to wt, d16–60 and d50–60. This increased biofilm formation is likely due to the alterations observed in surface polysaccharide, especially since surface polysaccharides are known to influence biofilm formation [34].

Based on these data, the deletions appear to have affected surface polysaccharide with no major role in environmental stress survival and minor changes to overall protein expression as observed by 2D-PAGE and secretome analysis. In fact, some of the differentially expressed proteins observed in d16–60 could be explained by changes to surface polysaccharide. These changes would most likely alter the permeability of the cell and when considering the interconnected nature of cell envelope structures, re-regulation of general porins (e.g. OmpU; spot 2CM in Table 2) and outer membrane proteins (OmpA; spot 1CM in Table 2) probably occurred to compensate. Consistent with this, outer membrane protein extractions also showed higher expression of an OmpU-like protein in d72–92 (data not shown). This change in permeability and subsequent re-regulation of porins may explain the minor variations in growth for all the deletion mutants. At this stage we cannot know how gene cassette products are altering host polysaccharide as further chemical characterisation of host polysaccharide is required, however, it is intriguing to hypothesise that gene cassette products modify or decorate host polysaccharide through addition of functional groups or sugars. A prior study had identified a gene cassette encoding an uncharacterised gene as important for CPS biosynthesis in a strain of *V. vulnificus* [35]. In the *V. rotiferianus* DAT722 array, there are some gene cassettes that suggest a role for polysaccharide modification or biosynthesis (Table S1). Cassette 31 contains a gene that encodes a putative β-phosphoglucomutase protein, a phosphotransferase that transfers a phosphate group to glucose and cassette 78 which encodes a putative O-acetyltransferase. Furthermore, there are numerous acetytransferases in the cassette array, four each span the deletions in d16–60 and d72–92. Research in our laboratory is underway to express such genes *in trans* to see whether they change the ^1^H NMR spectra and level of biofilm formation in the deletion mutants.

This study has answered important questions as well as advanced the integron biology field. Firstly, gene cassette products are highly novel and at this stage of research an attempt to identify phenotypes is difficult and relies on randomly selecting a phenotype to test. By determining that surface polysaccharide is a target of change in these deletion mutants, future research can be aimed at determining how gene cassettes modify surface polysaccharide. Secondly, this study conclusively demonstrated that gene cassettes do not need to be expressed from P_c_ to have an physiological impact and corroborates our previous study that identified numerous promoters in the *V. rotiferianus* DAT722 cassette array [9]. Thirdly, indel events are common in the cassette array of vibrios [5] indicating they have an important evolutionary and adaptive role. For the first time, we have shown how this affects *Vibrio* physiology by showing that a large deletion appears to largely affect surface polysaccharide. Future experiments are aimed at determining the biological ramifications of such changes by testing the deletion mutants in more biological assays such as biofilm formation to various substrata and bacteriophage assays.

### Conclusions

From this study we conclude that deletion of subsets of gene cassettes along the 116 cassette long array impacts on surface cell polysaccharide structures. How gene cassettes are altering polysaccharide structures requires further chemical analysis, however, any structural change to bacterial host polysaccharide is likely to impact on how the cell interacts with its environment and with other organisms within its environment.

## Materials and Methods

### Bacterial Strains and Growth Conditions

Bacterial strains and plasmids used in this study are listed in Table 1. *Vibrio* strains were routinely grown on Luria-Bertani medium supplemented with 2% NaCl (LB20) at 28°C. *Escherichia coli* strains were routinely grown on Luria-Bertani medium. Growth curves of all *Vibrio* strains were conducted in 24 well microtitre plates containing 1 ml of medium per well. The inoculum was from overnight cultures grown in LB20 and then diluted to OD_600_ of 0.7 using 2% NaCl. Growth curve cultures were inoculated at 1∶100 and growth measured using a microtitre plate reader (Synergy HT Bio-Tek) at OD_590_, and Gen5 (Bio-Tek) software. In experiments comparing growth of the wt and deletion mutants with different carbon sources, a marine minimal salts medium (2M) which mimics a seawater environment [36] was used supplemented with a carbon source (glucose at 11.1 mM and aspartic acid, succinate and fumarate at 20 mM respectively). Kanamycin was used at 100 µg/ml.

### DAT722 Cassette Analysis and Strain Construction

The cassette array of DAT722 is fully sequenced and consists of 116 gene cassettes although there are 94 different cassette types due to the presence of paralogous cassettes [37], [38]. Construction of the deletion mutants (Table 1) is as described previously [15]. Briefly, pMAQ1081 containing a 1834 bp fragment inserted into the *sacB*-counter selectable suicide vector pCVD442 [39] was used to create deletions in the cassette array of *V. rotiferianus* DAT722. The fragment consisted of two sequences with homology to different paralogous cassettes across the array disrupted with a kanamycin resistance gene. Conjugation of this construct into *V. rotiferianus* DAT722 allowed for allele replacement and deletion of cassettes between these two sets of paralogous cassettes. Deletion mutants d16–60 and d50–60 were created by taking a merodiploid (designated MD7) consisting of pMAQ1081 recombined into cassette 61 and screening colonies counter selected on sucrose medium with primers targeting unique cassettes outside the expected deletions. An identical approach was taken for creating d16–60a. An independently derived but identical merodiploid to MD7 (designated SC-8B61) was used. Deletion mutant d72–92 was isolated as a double-crossover and did not undergo sucrose counter selection. Deletion mutant d72–92a was created by taking a merodiploid (designated SC-8A91) consisting of pMAQ1081 recombined into cassette 93 and screening colonies counter selected on sucrose medium as described above.

### Stress Assays

*V. rotiferianus* DAT722 and deletion mutant were stressed with the following conditions as follows. All experiments were carried out in triplicate with data given in figures representative of the triplicate data.

#### Oxidative stress

1 mL of an overnight culture grown in LB20 was washed with 0.55×NSS and diluted 1∶10 in 0.55×NSS. The diluted culture was exposed to 0.5 mM hydrogen pyroxide with samples taken for enumeration at 0, 30 and 60 minutes post addition of H_2_O_2_.

#### Iron depletion stress

100 µl of an overnight LB20 culture was inoculated into 2M+glucose containing 0.1 mM of the iron chelating agent 2′2′,-dipyridyl (DP) and incubated at 28°C with shaking for 12 days. Samples were taken daily for enumeration of viable cells.

#### Cold shock

Cells were grown to mid-logarithmic phase in 2M+glucose (OD_600_ ∼ 0.3) and then placed at 4°C. Samples were taken daily for enumeration of viable cells.

### Differential Display Analysis of 2D-PAGE Gels

Overnight cultures of wt and mutant strains grown in LB20 and 2M+glucose were resuspended in 1 ml solution containing 1% C7bz0, 2M thiourea, 7M urea, 40 mM Tris and 50 mM LiCl. Resuspended samples were processed according to [40] and run on a 2D-PAGE gel in triplicate. Triplicate wt and mutant 2D-PAGE gels were analysed for post-translational modifications and up/down-regulations using software program PDQuest (Bio-Rad ver 8.0). Differentially displayed protein spots were cut out, trypsin digested and run through LC-MS/MS for identification at the UTS Protein and Proteomics Core Facility. LC-MS/MS data was run through PEAKS Studio software (Bioinformatics Solutions) in order to compare LC-MS/MS identified peptides to a protein output file acquired from the RAST annotated *V. rotiferianus* DAT722 genome [41].

### Supernatant Protein Extraction and Gel Electrophoresis

Cells were grown in 2M +0.2% glucose minimal media for 17 hrs at 28°C. Cells were collected by centrifugation (4000×*g*) and the supernatant was collected and filtered through a 0.2 µM filter. To precipitate supernatant proteins, five volumes of acetone was added, mixed by gentle inversion and incubated at −20°C for 30 mins. Precipitated proteins were collected by centrifugation at 3000×*g* for 3 mins and supernatant discarded and the pellet dried at 37°C overnight prior and total protein to weighing. The protein pellet was then resuspended to a concentration of 40 mg/ml in 2M thiourea, 7M urea and 1% C7bz0.

Prior to gel electrophoresis samples were run through a Micro-Biospin Column (Bio-Rad) according to their protocol to remove excess salt from the sample due to media. 400 µg of protein was loaded onto a 4–12% Bis/Tris precast polyacrylamide 1D gel (Bio-Rad) and run at 160V for ∼60 mins. Gels were then fixed for 30 mins in 10% acetic acid (v/v) and 40% methanol (v/v) prior to staining with Flamingo protein stain (Bio-Rad). Ladder used was Bio-Rad Precision Plus Protein Unstained Standard (catalog # 161-0363).

### Congo Red Staining of Bacterial Colonies

Wild-type *Vibrio rotiferianus* DAT722 and isogenic deletion mutants were plated out for single isolated colonies on LB20 and 2M +0.2% glucose plates containing 0.001% congo red (Sigma) and left for 7 days at 28°C. After 7 days colonies were observed on an Olympus SZX12 stereomicroscope at a magnification of 125× using a Colourview colour camera and images were collected using Image Analysis software (Olympus).

### Extraction of Whole Cell Polysaccharide

Whole cell polysaccharide of mutants grown in 2M +0.2% glucose overnight was extracted using a proteinase K, phenol-water method adapted from Apicella, 2008 [42]. For the ^1^H NMR scans of extracts shown in Figure 5, benzonase was used to remove contaminating DNA and RNA. For the ^1^H NMR scans of extracts shown in Figure S2, DNase I and RNase A was used to remove contaminating DNA and RNA.

### NMR Spectroscopy of Whole Cell Polysaccharide

Equal amounts of purified polysaccharide were exchanged three times using D_2_O as a solvent. 1D water suppression ^1^H NMR experiments were performed on purified whole cell polysaccharide resuspended in 600 µL of D_2_O using an Agilent Technologies 500 MHz NMR instrument at 28°C with the internal reference of the sodium salt of 3-(trimethylsilyl)-3,3,2,2-tetradeuteroproponic acid. The typical acquisition parameters utilized were spectral width 8012 Hz, acquisition time 4.089s, relaxation delay 1.5s and line boarding frequency 0.5 Hz.

### Biofilm/adhesion Assays

Overnight cultures of wt, d16–60, d72–92 and d50–60 grown in 2M+glucose were diluted 1∶100 and 500 µl added to the well on a flat bottom, hydrophilic, plastic 24-well microtitre plate (Nunc ThermoFisher) in quadruplet and incubated for 24 hrs at 28°C with shaking at 200 rpm. Quadruplet control wells were also set up containing media only. Unattached planktonic cells were then carefully removed by pipetting and wells were washed twice with 2% NaCl. 500 µl of crystal violet (0.2% w/v) was then added to wells and incubated for 15 mins to stain the adhered cells. The crystal violet was removed and the wells washed three times with 2% NaCl to remove excess stain. To measure the biomass of adhered cells, the crystal violet was solubilised with 30% (v/v) acetic acid, transferred to a clean microtitre plate and optical density (600 nm) measured using a Synergy HT plate reader (Bio-Tek). This experiment was repeated on three separate occasions.

## Supporting Information

Figure S1 **Isoelectric focusing contamination.** Triplicate 2D-PAGE gels of protein extracted from d16-60 cells grown in 2M+glucose to stationary phase. Horizontal streaking indicates disruption of the IEF stage due to a contaminating substance.
(TIF)

Figure S2 **Water suppression NMR spectra replicate.** H^1^ NMR spectra of wt, d16-60a and d72-92a whole cell polysaccharide.
(TIF)

Table S1 **DAT722 ordered cassette array.** List of 116 *V. rotiferianus* DAT722 gene cassettes with putative identification and putative conserved superfamily domains of proteins encoded by genes contained within the gene cassettes.
(DOCX)

Table S2 **Genome region encoding unknown protein found in 2D-PAGE.** *V. rotiferianus* DAT722 genome region containing gene encoding unknown protein identified in spot 1CS. The gene encoding this protein is the genomic region responsible for polysaccharide biosynthesis. Sequence found at accession # NZ_AFAJ01000014.
(TIF)
