# Supplementary material for: Deletion of Integron-Associated Gene Cassettes Impact on the Surface Properties of Vibrio rotiferianus DAT722
Source: PLoS One. 2013 Mar 6;8(3):e58430. doi: 10.1371/journal.pone.0058430 (PMC3590141; doi:10.1371/journal.pone.0058430)
Supplement: Table S1 — DAT722 ordered cassette array. List of 116 V. rotiferianus DAT722 gene cassettes with putative identification and putative conserved superfamily domains of proteins encoded by genes contained within the gene cassettes. (DOCX) [file pone.0058430.s003.docx]

| **Table S1: DAT722 ordered cassette array.** List of 116 *V. rotiferianus* DAT722 gene cassettes with putative identification and putative conserved superfamily.domains [[1](#_ENREF_1)] of proteins encoded by genes contained within the gene cassettes**.** | | | |
| --- | --- | --- | --- |
| **Cassette** | **Putative identification** | **# amino acids** | **Putative conserved superfamily domains** |
| 1 | Hypothetical protein | 200 | None |
| 2 | Phage-related protein | 277 | None |
| 3 | Hypothetical protein | 234 | None |
| 4 | FRG domain protein | 239 | FRG domain^1^ |
| 5 | ThiJ/PfpI family protein | 202 | GAT_1 domain^2^ |
| 6 | Hypothetical protein | 133 | None |
| 7 | Hypothetical protein | 93 | None |
| 8 | Hypothetical protein | 187 | None |
| 9 | Hypothetical protein | 153 | None |
| 10 | Hypothetical protein | 150 | None |
| 11 | DNA topoisomerase I | 237 | Mrr_cat domain^3^; zf-C4_Topoisom domain^4^ |
| 12 | Hypothetical protein | 188 | None |
| 13 | Hypothetical protein | 190 | None |
| 14 | Hypothetical protein | 79 | None |
| 15 | Hypothetical protein | 106 | None |
| 16 | Hypothetical protein | 190 | None |
| 17 | Hypothetical protein | 258 | None |
| 18 | Hypothetical protein | 208 | None |
| 19 | Hypothetical protein | 226 | DUF4145 domain^5^ |
| 20 | Antibiotic biosynthesis monooxygenase | 114 | ABM domain^6^ |
| 21 | MazG nucleotide phosphohydroloase | 94 | NTP-PPase domain^7^ |
| 22 | No significant coding region |  |  |
| 23 | Hypothetical protein | 119 | None |
| 24 | Acetyltransferase | 255 | NAT_SF domain^8^ |
| 25 | Hypothetical protein | 282 | None |
| 26 | No significant coding region |  |  |
| 27 | Hypothetical protein | 126 | None |
| 28 | Hypothetical protein | 214 | None |
| 29 | Putative restriction endonuclease | 289 | HNHc domain^9^ |
| 30 | Putative PAAR-containing motif | 176 | None |
| 31 | Phosphorylated carbohydrate phosphatase | 215 | HAD-like domain^10^ |
| 32 | Putative GNAT family acetyltransferase | 149 | NAT_SF domain^8^ |
| 33 | Hypothetical protein | 246 | None |
| 34 | No significant coding region |  |  |
| 35 | Hypothetical protein | 209 | None |
| 36 | No significant coding region |  |  |
| 37 | No significant coding region |  |  |
| 38 | Hypothetical protein | 139 | None |
| 39 | GNAT family acetyltransferase | 154 | NAT_SF domain^8^ |
| 40 | No significant coding region |  |  |
| 41 | Hypothetical protein | 159 | None |
| 42 | Hypothetical protein | 209 | None |
| 43 | Hypothetical protein | 218 | None |
| 44 | Hypothetical protein | 106 | DUF2834^11^ |
| 45 | Hypothetical protein | 223 | None |
| 46 | No significant coding region |  |  |
| 47 | Putative ribonuclease inhibitor | 93 | Barstar_like domain^12^ |
| 48 | No significant coding region |  |  |
| 49 | No significant coding region |  |  |
| 50 | No significant coding region |  |  |
| 51 | Hypothetical protein | 194 | None |
| 52 | Hypothetical protein | 105 | None |
| 53 | No significant coding region |  |  |
| 54 | Acetyltransferase | 165 | NAT_SF domain^8^ |
| 55 | No significant coding region |  |  |
| 56 | Hypothetical protein | 110 | None |
| 57 | Acetyltransferase | 151 | NAT_SF domain^8^ |
| 58 | Aminoglycoside phosphotransferase | 274 | PKc_like domain^13^ |
| 59 | DNA topology modulation protein | 165 | P-loop_NTPase domain^14^ |
| 60 | No significant coding region |  |  |
| 61 | No significant coding region |  |  |
| 62 | Hypothetical protein | 116 | None |
| 63 | No significant coding region |  |  |
| 64 | Retinol acyltransferase domain protein | 161 | LRAT domain ^15^ |
| 65 | hypothetical acetyltransferase | 142 | NAT_SF domain^8^ |
| 66 | No significant coding region |  |  |
| 67 | Hypothetical protein | 241 | None |
| 68 | Hypothetical protein | 78 | None |
| 69 | Histone acetyltransferase HPA2 | 156 | NAT_SF domain^8^ |
| 70 | Hypothetical protein | 118 | None |
| 71 | Hypothetical protein | 116 | None |
| 72A | Type VI secretion system-associated | 159 | DUF4285 domain^16^ |
| 72B | Hypothetical protein | 121 | None |
| 73 | No significant coding region |  |  |
| 74 | haemagglutinin associated protein | 224 | AdoMet-MTases^17^ |
| 75 | Hypothetical protein | 318 | TIR_2 domain^18^ |
| 76 | Hypothetical protein | 97 | None |
| 77 | Hypothetical protein | 91 | None |
| 78 | Maltose O-acetyltransferase | 195 | LbetaH domain^19^; Mac domain^20^ |
| 79 | Hypothetical protein | 246 | None |
| 80 | Hypothetical protein | 124 | None |
| 81 | Hypothetical protein | 102 | None |
| 82 | No significant coding region |  |  |
| 83 | No significant coding region |  |  |
| 84 | Acetyltransferase | 157 | NAT_SF domain^8^ |
| 85 | Hypothetical protein | 154 | GIY-YIG nuclease domain^21^ |
| 86 | Hypothetical protein | 157 | None |
| 87 | No significant coding region |  |  |
| 88 | Acetyltransferase | 173 | NAT_SF domain^8^ |
| 89 | Hypothetical protein | 102 | None |
| 90 | No significant coding region |  |  |
| 91 | Acetyltransferase | 173 | NAT_SF domain^8^ |
| 92 | Hypothetical protein | 102 | None |
| 93 | No significant coding region |  |  |
| 94 | Hypothetical protein | 292 | None |
| 95 | Hypothetical | 238 | None |
| 96 | No significant coding region |  |  |
| 97 | Cold shock protein | 155 | S1_like domain^22^;Excalibur calcium-binding domain^23^ |
| 98 | Hypothetical protein | 210 | None |
| 99 | Hypothetical protein | 318 | None |
| 100 | No significant coding region |  |  |
| 101 | Hypothetical protein | 289 | none |
| 102 | Toxin-antitoxin plasmid stability protein | 107 | Plasmid stabilisation system domain^24^ |
| 103 | Acetyltransferase | 143 | NAT_SF domain^8^ |
| 104 | Hypothetical protein | 152 | Transglut_core domain^25^ |
| 105 | No significant coding region |  |  |
| 106 | Hypothetical protein | 127 | None |
| 107 | Glyoxalase/bleomycin resistance protein/dioxygenase protein | 117 | Glo_EDI_BRP_like domain^26^ |
| 108 | Hypothetical protein | 244 | None |
| 109 | Restriction endonuclease-like protein | 264 | HNHc domain^9^ |
| 110 | Putative phage-related membrane protein | 155 | None |
| 111 | Hypothetical protein | 100 | None |
| 112 | Putative cytoplasmic protein | 172 | DUF2778 domain^27^ |
| 113 | Hypothetical | 122 | None |
| 114 | Cytoplasmic protein | 161 | DUF4285 domain^15^ |
| 115 | No significant coding region |  |  |
| 116 | Hypothetical protein | 149 | None |

^1^ This presumed domain contains a conserved N-terminal (F/Y)RG motif. It is functionally uncharacterised.

^2^ Type 1 glutamine amidotransferase (GATase1)-like domain.

^3^ Prokaryotic family found in type II restriction enzymes containing the hallmark (D/E)-(D/E)XK active site. Presence of catalytic residues implicates this region in the enzymatic cleavage of DNA.

^4^ Topoisomerase DNA binding C4 zinc finger.

^5^ This domain is found in a variety of restriction endonuclease enzymes. It is functionally uncharacterised.

^6^ This domain is found in monooxygenases involved in the biosynthesis of several antibiotics by Streptomyces species.

^7^ This superfamily contains enzymes that hydrolyze the alpha-beta phosphodiester bond of all canonical NTPs into monophosphate derivatives and pyrophosphate (PPi).

^8^ N-Acyltransferase superfamily: Various enzymes that characteristically catalyse the transfer of an acyl group to a substrate.

^9^HNH endonuclease signature which is found in viral, prokaryotic, and eukaryotic proteins.

^10^ The haloacid dehalogenase (HAD) superfamily includes carbon and phosphorus hydrolases. These proteins catalyse nucleophilic substitution reactions at phosphorus or carbon centres, using a conserved Asp carboxylate in covalent catalysis.

^11^ Protein of unknown function (DUF2834) ;This is a bacterial family of uncharacterised proteins.

^12^Barstar is an intracellular inhibitor of barnase, an extracellular ribonuclease of Bacillus amyloliquefaciens. Barstar binds tightly to the barnase active site and sterically blocks it, thus inhibiting its potentially lethal RNase activity inside the cell.

^13^Protein Kinases, catalytic domain ;The protein kinase superfamily is mainly composed of the catalytic domains of serine/threonine-specific and tyrosine-specific protein kinases.

^14^P-loop containing Nucleoside Triphosphate Hydrolases ;Members of the P-loop NTPase domain superfamily are characterized by a conserved nucleotide phosphate-binding motif.

^15^Lecithin retinol acyltransferase ;The full-length members of this family are representatives of a novel class II tumour-suppressor family.

^16^ This family of proteins is functionally uncharacterised.

^17^S-adenosylmethionine-dependent methyltransferases (SAM or AdoMet-MTase), class I; AdoMet-MTases are enzymes that use S-adenosyl-L-methionine (SAM or AdoMet) as a substrate for methyltransfer, creating the product S-adenosyl-L-homocysteine (AdoHcy).

^18^This is a family of bacterial Toll-like receptors.

^19^Left-handed parallel beta-Helix (LbetaH )domain: The alignment contains 5 turns, each containing three imperfect tandem repeats of a hexapeptide repeat motif .Proteins containing hexapeptide repeats are often enzymes showing acyltransferase activity, however, some subfamilies in this hierarchy also show activities related to ion transport or translation initiation.

^20^Maltose acetyltransferase ;This domain family is found in bacteria, archaea and eukaryotes, and is approximately 50 amino acids in length. Mac uses acetyl-CoA as acetyl donor to acetylated cytoplasmic maltose.

^21^The GIY-YIG nuclease domain superfamily includes a large and diverse group of proteins involved in many cellular processes, such as class I homing GIY-YIG family endonucleases, prokaryotic nucleotide excision repair proteins UvrC and Cho. All of these members contain a conserved GIY-YIG nuclease domain that may serve as a scaffold for the coordination of a divalent metal ion required for catalysis of the phosphodiester bond cleavage.

^22^Ribosomal protein S1-like RNA-binding domain. Found in a wide variety of RNA-associated proteins. Originally identified in S1 ribosomal protein. This superfamily also contains the Cold Shock Domain (CSD), which is a homolog of the S1 domain. Both domains are members of the Oligonucleotide/oligosaccharide Binding (OB) fold.
^23^Extracellular Ca2+-dependent nuclease YokF from Bacillus subtilis and several other surface-exposed proteins from diverse bacteria are encoded in the genomes in two paralogous forms that differ by a ~45 amino acid fragment, which comprises a novel conserved domain.

^24^ Plasmid stabilisation system protein ;Members of this family are involved in plasmid stabilisation. The exact molecular function of this protein is not known. This family also encompasses RelE/ParE . RelE/StbE family ;Plasmids may be maintained stably in bacterial populations through the action of addiction modules, in which a toxin and antidote are encoded in a cassette on the plasmid. In any daughter cell that lacks the plasmid, the toxin persists and is lethal after the antidote protein is depleted. Toxin/antitoxin pairs are also found on main chromosomes, and likely represent selfish DNA.

^25^This family includes animal transglutaminases and other bacterial proteins of unknown function.

^26^This domain superfamily is found in a variety of structurally related metalloproteins, including the type I extradiol dioxygenases, glyoxalase I and a group of antibiotic resistance proteins.

^27^Protein of unknown function (DUF2778) ;This is a bacterial family of uncharacterised proteins.

**References**

1. Marchler-Bauer A, Zheng C, Chitsaz F, Derbyshire MK, Geer LY, et al. (2013) CDD: conserved domains and protein three-dimensional structure. Nucl Acids Res 41: D348-D352.
