# Supplementary figures and images for: Deletion of Integron-Associated Gene Cassettes Impact on the Surface Properties of Vibrio rotiferianus DAT722
Source: PLoS One. 2013 Mar 6;8(3):e58430. doi: 10.1371/journal.pone.0058430 (PMC3590141; doi:10.1371/journal.pone.0058430)

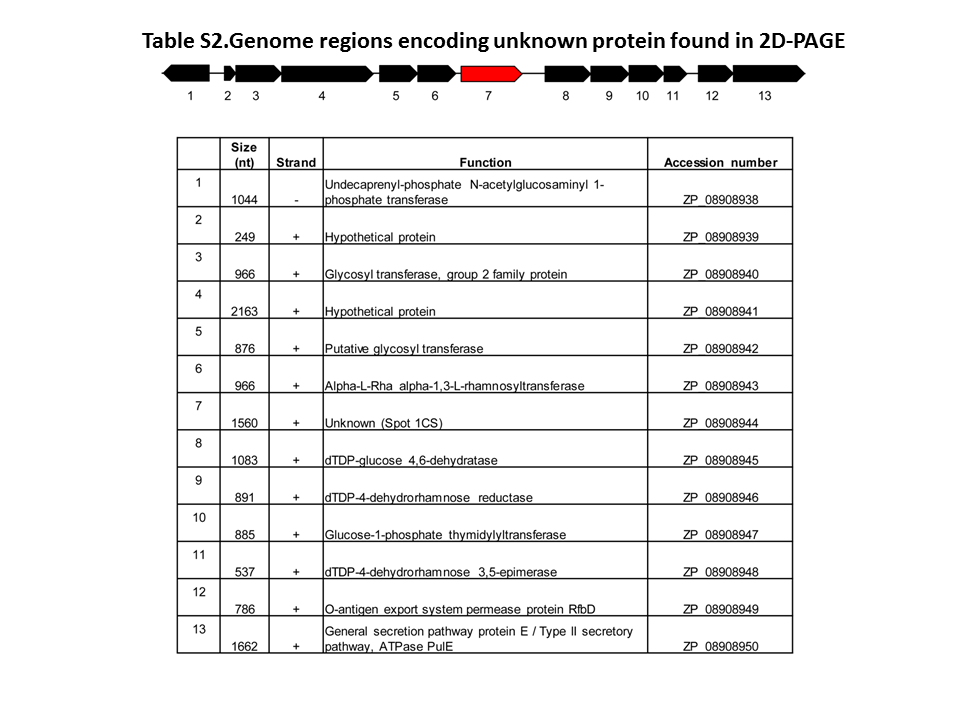

Supplement: Table S2 — Genome region encoding unknown protein found in 2D-PAGE. V. rotiferianus DAT722 genome region containing gene encoding unknown protein identified in spot 1CS. The gene encoding this protein is the genomic region responsible for polysaccharide biosynthesis. Sequence found at accession # NZ_AFAJ01000014. (TIF) [file pone.0058430.s004.tif]
